# Survival Rates of Reinserted Orthodontic Microimplants: An Exploratory Systematic Review

**DOI:** 10.3390/jcm15093489

**Published:** 2026-05-02

**Authors:** Kacper Galant, Maja Podziewska, Norbert Soboń, Natalia Turosz, Konrad Małkiewicz

**Affiliations:** 1 Independent Researcher, 92-213 Lodz, Poland; kacpergalant.ld@gmail.com; 2 Student Scientific Club, Department of Orthodontics, Medical University of Lodz, 251, Pomorska St., 92-213 Lodz, Poland; maja.podziewska@stud.umed.lodz.pl (M.P.);; 3 National Medical Institute of the Ministry of the Interior and Administration, 137, Wołoska St., 02-507 Warsaw, Poland; 4 Department of Maxillofacial Surgery, Hospital of the Ministry of the Interior and Administration, Wojska Polskiego 51, 25-375 Kielce, Poland; 5 Department of Orthodontics, Medical University of Lodz, 251, Pomorska St., 92-213 Lodz, Poland

**Keywords:** orthodontic anchorage procedures, replantation, dental implants, treatment outcome, bone density, palate, hard, review

## Abstract

**Background/Objectives**: The loss of orthodontic microimplants is a common clinical complication that significantly disrupts the continuity of malocclusion treatment. Despite increasing clinical use of microimplant reinsertion, the factors influencing its success remain unclear. The aim of this exploratory systematic review was to examine the available literature regarding clinical outcomes related to the retention of orthodontic microimplants following their reinsertion. **Methods**: Studies that assessed the success of orthodontic microimplant reinserted were included in the review. Searches were conducted on 27 September 2025, in the following databases: BASE (Bielefeld Academic Search Engine), PubMed, Scopus, and EMBASE. The ROBINS-I (Risk of Bias in Non-randomized Studies—of Interventions) tool was used to assess the risk of bias. Due to heterogeneity of included studies, a narrative synthesis was performed. **Results**: Four of the 577 studies were included in the review. A total of 305 microimplants were reinserted in 276 patients. The overall success rate ranged from 44.16% to 66%. Analysis indicated a significantly higher success rate in the maxilla (up to 68.60%) compared to the mandible (lowest 36.84%). Furthermore, a narrative synthesis suggests better clinical outcomes for 8 mm long microimplants compared to 6 mm ones, as well as reduced reinsertion success in areas with high cancellous bone density. Regarding the modification of the insertion site, the current data are contradictory; while some studies indicate significant benefits from changing the site (e.g., to the midpalatal suture), others show no statistical difference compared to reinsertion at the same site. Overall, the evidence remains limited and heterogeneous. **Conclusions**: The current review of the literature on the success of reinsertion of orthodontic microimplants is subject to a high risk of misinterpretation, due to the limited amount of data and the risk of unidentified confounding factors. Further standardized clinical trials are needed to develop unified protocols for these procedures. **Other**: The review was prospectively registered with the Open Science Framework (OSF); osf.io/tbj2s.

## 1. Introduction

### 1.1. Rationale

Orthodontic anchorage is resistance to unwanted tooth movement [1,2]. Orthodontists who prefer traditional methods of increasing this resistance typically use extraoral anchorage (headgear or facemask) and intraoral anchorage, such as the translingual or transpalatal arch, or Nance’s plate [1,3]. One of the increasingly widely implemented solutions is skeletal anchorage, which minimizes unwanted displacement of individual teeth or tooth groups [4,5]. Although this method is becoming increasingly common, it still presents a challenge for a significant number of orthodontists, and certain technical aspects of using orthodontic microimplants still raise doubts [6].

Microimplants, similarly to ankylosed teeth, enable attachment of orthodontic appliances, or their components ensuring they remain stable and immobile under orthodontic forces [7,8]. This prevents so-called loss of anchorage during treatment, i.e., tooth movement caused by applied forces that are not the goal of the treatment. These include, in particular, uncontrolled loss of post-extraction space, undesirable movement of anterior teeth during distalization of posterior teeth, and extrusion of correctly positioned teeth during intrusion of teeth extending beyond the occlusal plane [9,10].

Orthodontic microimplants are devices temporarily inserted into the patient’s bone during treatment, enabling direct (attachment of an orthodontic appliance component) or indirect (bonding a tooth to a ligature) stabilization of individual teeth or groups of teeth. They are inserted intraorally into the maxillary alveolar process, the mandibular alveolar region, or the palatal suture area, after prior radiological assessment of selected anatomical regions [4,5,6]. The primary clinical indications for the placement of orthodontic microimplants include absolute anchorage reinforcement, en-masse retraction of anterior teeth, molar distalization, and intrusion of overerupted teeth [1,9,10]. Due to their small dimensions, highly biocompatible titanium microimplants can be inserted in a wide range of maxillary and alveolar bone sites [1,4,10]. However, their placement has clear contraindications, such as active periodontal inflammation, poor oral hygiene, insufficient bone volume at the planned insertion site, and certain systemic metabolic bone diseases that compromise osseointegration or primary stability [3,5,11,12].

Unfortunately, microimplant loss is a common complication during orthodontic treatment. This phenomenon is typically attributed to improper implant site selection, errors in surgical technique, excessively rapid or inappropriate mechanical loading of the implant, or individual patient response, correlating with innate mechanical strength limitations [2,13].

Microimplant failure necessitates a reassessment of the treatment strategy. Clinicians face several distinct options: complete removal of the device without replacement (resection), delayed application of orthodontic forces on an already placed but temporarily destabilized implant (reloading), or the surgical placement of a new microimplant to continue skeletal anchorage (reinsertion). For the purpose of this review, reinsertion is defined strictly as the placement of a new orthodontic microimplant following the failure and removal of a primary implant, regardless of whether the secondary placement occurs in the exact same anatomical site (orthotopic) or an adjacent alternative location (ectopic). Among these, reinsertion represents the primary focus of the present review. Surgical procedures typically involve modifying the implant site and inserting larger implants, especially if implantation is in a similar anatomical location to the previous one. To date, no consensus has been reached on a strict protocol for this procedure. Therefore, to prevent the risk of repeated implant loss, it is important to analyze the factors affecting implant retention after reinsertion. Particular attention is given to anatomical factors, implant characteristics, and procedural variables that may influence the success of reinsertion.

### 1.2. Objectives

Despite the high frequency of primary microimplant loss and the common clinical necessity of their reinsertion, there is a significant lack of systematized evidence determining the factors that condition secondary clinical success. This lack of comprehensive data and unified clinical protocols regarding microimplant reinsertion constitutes a critical knowledge gap in contemporary orthodontics. The aim of this study is to systematically review the available literature regarding clinical variables related to orthodontic microimplant retention following reinsertion during malocclusion therapy.

## 2. Methods

### 2.1. Protocol and Registration

The protocol for this review was prospectively published in the Open Science Framework Registries (https://osf.io/tbj2s; published 15 September 2025). This systematic review was conducted and reported in accordance with the PRISMA guidelines (Files S1 and S2).

### 2.2. Eligibility Criteria

The review includes studies addressing situations in which a primary microimplant loss occurred followed by the decision to maintain skeletal anchorage during treatment and reinsert an orthodontic microscrew.

Detailed eligibility criteria are presented in Table 1.

### 2.3. Information Sources and Search Strategy

The most popular scientific literature databases were searched on 27 September 2025: (1) Bielefeld Academic Search Engine (BASE), (2) PubMed, (3) Scopus, and (4) EMBASE. The search query was created by K.G. and K.M. It utilized synonyms for orthodontic microimplants—including Temporary Anchorage Devices (TADs)—and additional keywords: procedure success and microimplant reinsertion. The final query is presented below:

(orthodontic OR orthodontics) AND (miniscrew OR miniscrews OR microimplant OR microimplants OR tad OR miniimplant OR anchorage) AND (reinsertion OR reimplantation OR reuse OR secondary OR repeat) AND (stability OR success OR survival OR risk OR outcome).

To ensure consistency in the search for studies, a unified search strategy was used across all databases. Given the exploratory nature of the review and the limited number of relevant studies, a broad and sensitive search approach was prioritized over database-specific optimization.

### 2.4. Selection Process

The evaluation of included publications was conducted in two stages. After manual deduplication by M.P., the studies were selected for further analysis based on their titles and abstracts, followed by full-text evaluation. The entire process was conducted by two authors (K.G. and M.P.). In case of doubt, the third author (K.M.) had the final say. Inter-rater agreement was assessed using the Cohen’s kappa coefficient, calculated using MedCalc (Version 23.0.2; MedCalc Software Ltd., Ostend, Belgium). The inter-rater agreement was K = 0.75, corresponding to good compliance. In the event of disagreement after the evaluation of titles and abstracts, the paper was moved to the next stage and assessed in full text using the previously established eligibility criteria. The Rayyan tool (Version 2024.08.29; Rayyan Systems Inc., Cambridge, MA, USA) was used for the selection process.

### 2.5. Data Collection Process and Data Items

Data from the included scientific publications were extracted by two authors (K.G. and N.S.) and presented in tables.

The following data were collected: (1) patient characteristics, (2) overall success of reoperation, (3) success based on implant location, (4) success based on whether or not repositioning was performed, (5) success based on implant characteristics, and (6) success based on the timing of microimplant reinsertion. These definitions and a more detailed discussion are provided in Table 2 for easier reader understanding.

### 2.6. Study Risk of Bias Assessment

The authors assessed the methodological behavior in eligible articles using the Risk Of Bias In Non-randomized Studies—of Interventions (ROBINS-I) tool. This tool comprises seven criteria, including assessment of confounding, selection of participants and reported results and classification of interventions. Moreover, deviations from intended interventions, missing data and measurement of outcomes were analyzed. To increase the transparency of the assessment, a traffic light plot and summary plot were created illustrating the judgments for each included study across all domains using the Robvis Tool [14].

### 2.7. Effect Measures and Synthesis Methods

Data from individual studies were presented in tables. For selected variables, the number of successful implants and the number of all implants were reported.

As noted in Table 2, the definition of ‘success’ varied across the included studies (ranging from stability for 8 months to stability until treatment completion). Due to the limited number of available studies (*k* = 4) and the substantial clinical and methodological heterogeneity regarding these follow-up horizons, performing a quantitative meta-analysis or meta-regression was deemed inappropriate. Pooling such heterogeneous outcomes would violate core assumptions of meta-analysis and risk producing clinically misleading estimates. Therefore, a narrative synthesis was conducted. The extracted data were narratively summarized to highlight clinical trends, evaluate potential factors associated with the success of orthodontic microimplant reinsertion, and identify gaps in the current literature.

## 3. Results

### 3.1. Study Selection

**Figure 1:**
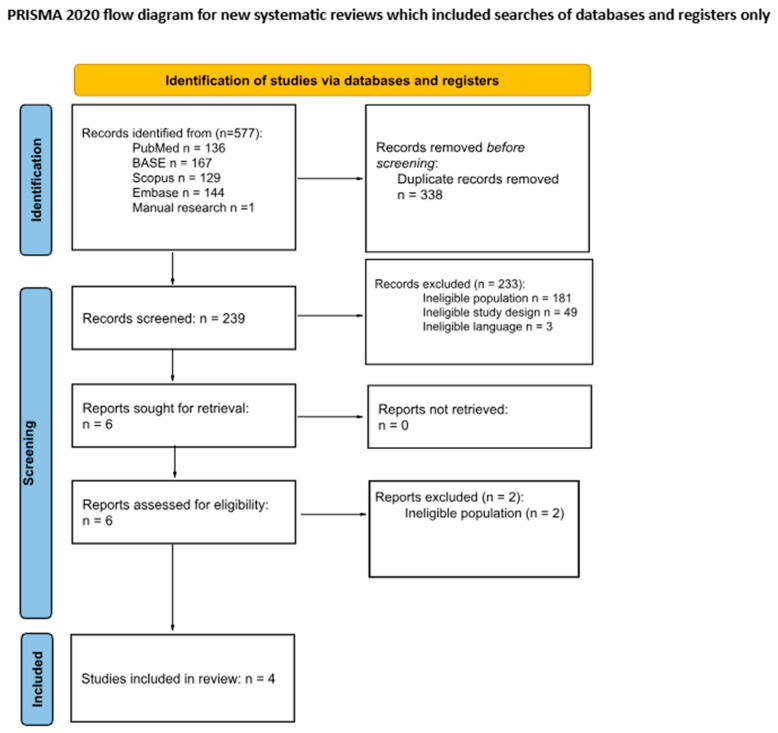
Flow diagram.

The initial search included 577 records: 136 from PubMed, 167 from BASE, 129 from Scopus and 144 from Embase, and the other 1 was located through manual searching. A total of 338 articles were removed before screening due to duplication. The authors screened 239 articles, of which 4 were jointly qualified [6,15,16,17]. At this stage, 233 records were excluded for the following reasons: ineligible population (*n* = 181), ineligible study design (*n* = 49), and ineligible language (*n* = 3). The vast majority of records excluded due to an ineligible population were studies evaluating the primary success rates of orthodontic microimplants in general orthodontic patients. Therefore, 2 reports were excluded at the full-text stage (Table A3), leaving 4 studies included in the final review (Figure 1).

Details of this process are presented in Figure 1.

### 3.2. Study Characteristics

The issue of orthodontic microimplant reinsertion is a topic rarely assessed in the available literature. Over the past 17 years, only four studies have been published discussing the success of microimplant reinsertion.

A total of 305 implants were reinserted in a group of 276 patients (Table 3). In all studies, the chosen procedure was flapless surgery with self-drilling. Table A1 presents detailed data on the implant systems and procedures used. Given the small number of available studies, along with the inherent confounding factors, inconsistent definitions of success, and methodological heterogeneity between them, the obtained data allow only for an exploratory overview rather than a definitive summary assessment of the success rate of this procedure.

### 3.3. Risk of Bias Within Studies

**Figure 2:**
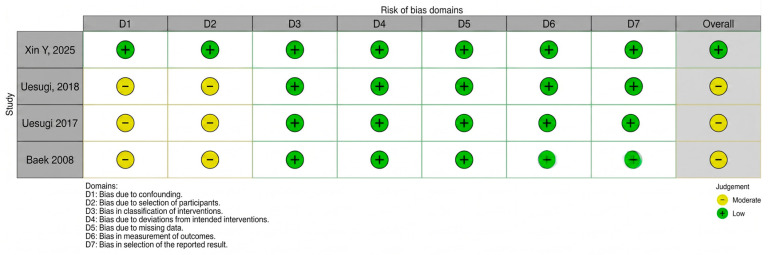
A graphical representation of evaluations at the domain level for each individual study [6,15,16,17].

**Figure 3:**
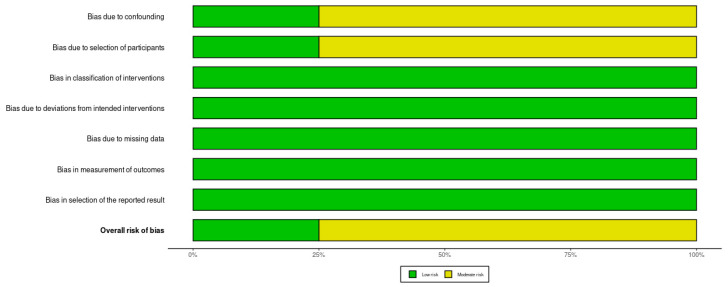
Bar visualizations representing the distribution of risk-of-bias evaluations across each domain.

Of the four included articles, three were assessed as having a moderate risk of bias (Figure 2 and Figure 3) [15,16,17]. This assessment was primarily due to the moderate risk of bias in domain D1 (confounding factors) and D2 (bias in selection of participants). The authors of these studies did not account for important factors such as bone density and quality, smoking, or patient hygiene, which could influence the procedure. Furthermore, the risk of bias in participant selection (Domain D2) was downgraded to moderate for the retrospective cohort studies (Uesugi 2017, Uesugi 2018, Baek 2008) [15,16,17]. In these settings, the inclusion of patients was based on the prior failure of their primary microimplants without rigorous randomization, inherently introducing selection bias. In contrast, only the study by Xin et al. was found to have a low risk of bias [6]. The authors of this publication were the only ones to measure and statistically account for key confounding factors, specifically cancellous and cortical bone density measured using CBCT. This paves the way to further research and refinement of the framework of factors affecting orthodontic microimplant treatment success. All observed associations should be interpreted as hypothesis-generating due to moderate risk of bias and lack of confounder control in most included studies.

### 3.4. Results of Individual Studies and Results of Syntheses

The overall success rate (SR ± SD) of reinsertion was a maximum of 63.83% [15] and a minimum of 44.16% [16]. In all analyzed studies, each author compared the SR of microimplants initially placed in the bone to the SR of reinserted screws. Only Baek et al. reported no statistical difference between the success of primary and secondary implant insertion [17]. It is important to note here the different criteria for successful implant retention. Both studies by Uesugi et al. considered success as retention of implant stability one year after insertion, along with the absence of signs of inflammation in the surrounding tissues [15,16]. In the study by Baek et al., this period was shorter (at least 8 months) [17]. Xin et al. defined success as the retention of stability until the end of treatment [6].

Each study analyzed a number of factors that could influence the obtained results. These included location in the mandible or maxilla (Table 4), change in implant site (Table 5), MI length (Table 6), MI diameter (Table 7), time from explantation to reinsertion (Table 8), and precise location relative to anatomical landmarks (Table A2). Table A1 additionally presents the manufacturers of the implant systems used in the studies, as this factor could also have influenced the presented results, although it was not analyzed in the included studies. Uesugi (2017) demonstrated a significantly higher reinsertion success rate in the maxilla compared to the mandible (*p* < 0.05) [16]. Xin (2025), however, did not confirm significant differences between the maxilla and mandible, although a greater cortical bone thickness in the mandible was noted, which could have contributed to slightly higher stabilization rates [6]. Both Uesugi and Xin emphasized the importance of bone quality for reinsertion stability [6,15,16]. More recent reports by Uesugi highlighted the difference in SR by comparing MI placement in the molar buccal area versus the midpalatal suture area [15]. This time, significant differences were demonstrated during reinsertion, favoring the palatal location, which was not reflected in the analysis of the initial implantation, where no statistical differences were observed between these groups. An additional factor considered by the authors was the change in MI location relative to the initial insertion site. The results obtained by Uesugi 2018 showed a significant increase in success after changing the reinsertion site from the molar buccal area to the midpalatal suture area (*p* < 0.05), while the Uesugi 2017 study did not confirm this correlation, indicating no difference in SR between reinsertion in the same and different sites [15,16]. However, the other two studies did not observe such a relationship, despite mean differences in SR. The study by Xin et al. was the only one to analyze bone density at the implant site, and demonstrated a significant decrease in MI retention with increasing cancellous bone density [6]. Xin et al. also assessed the influence of cortical plate thickness (CT), total bone thickness (BT), and soft tissue type at the implant site [6]. Although none of these parameters showed a significant effect on SR, the authors noted a tendency toward lower stability of implants placed in areas with thinner and more mobile mucosa. According to descriptive data, the highest success rate was observed in the midpalatal suture area, and the lowest in the posterior mandibular area. The available data regarding the rationale for changing the implantation site are contradictory. Although some studies indicate significant benefits from relocating the implant (e.g., to the midpalatal suture), others show no statistical differences, meaning this issue remains genuinely unresolved and requires further investigation. Another factor considered was the length and width of the implant used. In the Uesugi 2018 study [15], length and diameter data were available for only 75 of the 94 reinserted screws (excluding screws reinserted in new locations), therefore the totals in the tables do not correspond to the total number of implants studied. A significant difference was observed between 6 mm and 8 mm microimplants [15,16] in favor of the longer MI. Compared to 8 mm and 10 mm lengths, the differences were marginal [6]. Each of the analyzed studies presented insufficient data for analyzing the effect of microimplant diameter on SR due to the lack of directional analysis of different diameters with the same lengths.

Regarding general factors related to patient age, gender, and health, the results are also contradictory. According to Xin et al., stabilization was significantly lower among younger patients [6]. Baek et al. reported contrary results; in their study, gender had a significant effect—men had a higher success rate, a finding not confirmed in any of the other studies analyzed [17]. This analysis also contrasts with the primarily inserted screws evaluated in the same study, where the SR was higher in women. The studies emphasize the importance of patient hygiene and habits (smoking, eating), but their impact was not directly examined. An interesting analysis by Baek et al. indicates a higher success rate for reinserted MIs in patients with Angle Class III compared to Classes I and II, which is opposite of the results for primarily inserted implants [17].

The final factor analyzed that may influence the SR of reinserted orthodontic microimplants was the time elapsed between reinsertion and implant loading (Table 8). Uesugi and Baek were the only authors to examine the immediate loading protocol [15,17]. It should be noted that immediate loading in the Uesugi study was used only for implants in the midpalatal suture area, which excludes direct comparison with the results of Baek et al. [15,17]. All of the analyzed studies were characterized by a lack of randomization of implant length or diameter, as they were individually selected based on patient anatomical conditions.

## 4. Discussion

### 4.1. Discussion of Systematic Review Findings

Despite the inclusion of four articles describing the results of studies evaluating a total of over 300 inserted orthodontic microimplants, it is impossible to draw clear conclusions or present a universal clinical protocol for the implant reinsertion procedure based on the available material. Most importantly, none of the included studies specifically analyzed the etiology of primary failure, which is likely a key prognostic factor for reinsertion success. One of the major limitations of all the included studies is the lack of randomization, as the implantation site and screw size were individually selected based on the patient’s condition, anatomy, and the treated defect.

Baek was the only one to note that the SR of implant reinsertion was significantly higher in patients with Class III malocclusion compared to patients with Class I and II malocclusion [17]. This factor was not considered by the authors of the remaining studies. Due to the limited and heterogeneous nature of the available data, it was not possible to observe any clear associations between the success of repeated surgical procedures and implant diameter or time to reloading. Inconsistent data regarding the location of orthodontic microimplants and general factors influencing the success of reinsertion limit the ability to synthesize data and draw consistent conclusions.

According to the cited authors, the implant type and insertion site were individually adapted to each clinical situation, further limiting the ability to clearly assess the impact of individual procedural parameters on SR. A recurring hypothesis generated by these authors is that avoiding reinsertion in the same location after primary failure and considering alternative sites, such as the palatal suture, while simultaneously using longer screws, might improve stability. However, this remains an observation rather than an established clinical protocol. Although a slightly higher success rate was observed when the implantation site was changed, a more detailed analysis of the success of site modification and reinsertion in the same location is difficult because in Uesugi’s study, site changes were not random, and some implants were deliberately relocated to the palatal suture area [15]. Literature regarding primary implantations suggests that sites covered by attached mucosa might offer more favorable conditions [18,19,20], though robust statistical confirmation specifically for reinsertion procedures is currently lacking due to data limitations.

In the analyzed studies, descriptive data suggest a more favorable clinical outcome for 8 mm implants compared to 6 mm implants, which exhibited highly variable success rates across the reports. This observation aligns with the clinical trend initially reported by Uesugi, although it still warrants rigorous investigation in future controlled trials. Generally, it can be assumed that MI reinsertion is a procedure with a lower success rate than primary implantation. This is contradicted by the study by Baek, who found no such differences. It should be emphasized that this study included the smallest sample size, which reduces the reliability of the results. Regarding implant diameter, 2 mm microimplants demonstrated the highest descriptive success rates. However, the studies did not use MIs of different lengths with the same diameter; therefore, this trend, although promising, does not allow for drawing clear conclusions. The extremely small number of available studies evaluating this specific parameter limits the ability to confirm these observations, serving rather as an implication for further research aimed at evaluating emerging trends.

Differences in MI reinsertion success depending on the insertion site can be described as inconsistent based on the analyzed studies. This is a key point requiring further discussion. The most thorough analysis of bone quality was conducted by Xin et al., and the results suggest that denser cancellous bone reduces reinsertion success, negating the significance of cortical bone thickness [6]. This is consistent with the lower success rate observed in the analysis for younger patients, who are characterized by a greater amount of hard cancellous bone. These results are consistent with those observed for primary surgical procedures [7,8,21,22,23]. The reduced effectiveness of orthodontic microimplants in adolescent patients may also result from their faster metabolism, which hinders the process of osseointegration with the surrounding hard tissues [8]. Furthermore, another factor that may affect the success of the procedure, related to the young age of patients, is their lower health awareness compared to adults, including their attention to dental health and adherence to hygiene recommendations. It is common knowledge that neglect in this regard contributes to a higher risk of failure in oral surgical procedures, which was proven in numerous studies [11,12,24,25].

Both analyses conducted by Uesugi, that considered the influence of bone conditions on implant success highlighted the fact that the presence of a thicker cortical bone plate, such as that found in the palatal suture area, increased the likelihood of success compared to other anatomical locations [15,16].

At the same time, the lowest success rate for implant procedures was observed in the lateral jaw region. In the study conducted by Xin, the author points to the problem of dense cancellous bone [6]. It is therefore possible that the success of the surgical procedure depends on the appropriate combination of both types of substrate; thick cortical bone provides high primary stability (which is crucial, as suggested by Uesugi), while less dense cancellous bone (as indicated by Xin) may provide better vascularization and regenerative potential at the site of healing after primary failure.

Dense cancellous bone may also be more susceptible to overheating during re-drilling, which ultimately leads to bone necrosis and implant loss. The authors suggest the palatal suture area as an alternative implant site in the event of failure of the initial procedure. It is important to emphasize the difference in success rates between procedures performed in the maxilla and mandible. These findings align with recent literature regarding primary implantation, which also emphasize the important role of cortical bone thickness and the biomechanical advantages of palatal placement for temporary anchorage devices [26,27].

This phenomenon is also likely related to the quality of the underlying bone. Further studies analyzing bone thickness and density at the implant site may help clarify these differences.

The authors of the included studies used different guidelines regarding the loading time of implants after insertion.

Baek et al. reported a general range of 2 to 3 weeks [17]. Uesugi et al. (2017), on the other hand, loaded MI immediately, extending this time to 3 months in some cases [16]. In the second study by Uesugi et al., and the study described by Xin et al., the authors did not specify this parameter at all [6,15]. The cited authors reported no clear evidence of an association between the time of loading initiation and implant mobility; therefore [28,29,30], they cautiously applied an immediate loading protocol. The cited authors point to primary stability as sufficient for transferring orthodontic forces. Furthermore, variations in surgical technique—such as the applied insertion torque and the choice between self-drilling or pre-drilling protocols—likely play a crucial role in secondary stability. However, because these specific biomechanical parameters were not uniformly reported or isolated in the included studies, their direct impact on reinsertion success remains an uncontrolled confounding factor. This highlights the need for an individual case assessment in terms of treatment needs, orthodontic mechanics, underlying bone anatomy, general health status and level of health awareness.

A significant limitation of the studies included in this review is the variation in the implant systems used (Table A1). This lack of standardization—including unmeasured differences in thread design, taper, and specific alloy properties—significantly hinders the direct comparison of clinical outcomes between individual studies and acts as a major confounding factor in evaluating reinsertion success rates.

However, it is worth noting that all cited authors used orthodontic microimplants made of a titanium alloy (Ti-6Al-4V), which remains the material of choice [31], characterized by good mechanical properties and biocompatibility.

### 4.2. Review Limitations and Directions for Future Research

A critical methodological limitation of this systematic review is the variability in how the primary authors defined clinical ‘success’. The narrative synthesis evaluated outcomes based on different follow-up horizons, including stability for 8 months, 1 year, and until treatment completion. Because the follow-up periods are not uniform, the reported success rates may not accurately reflect long-term stability and could over- or underestimate the true effectiveness of the reinsertion procedure depending on the intended duration of anchorage required for a specific clinical case. Future studies must employ standardized definitions of success and clearly report survival rates at uniform time intervals to allow for more robust statistical comparisons.

The systematic review was conducted using a broad search query, utilizing the most popular databases. No automated time or language filters were applied during the database searches. However, three studies were excluded (as shown in Figure 1) because they were published in languages not comprehended by the authors and lacked full English translations. Due to the high success rate of the initial procedure, there are few publications evaluating microimplant reinsertion procedures, which significantly limits the strength of the clinical evidence and the scope of this narrative synthesis.

Despite these limitations, the review addresses a very important and timely clinical issue. Because the included studies were non-randomized and the choice of implant characteristics was dictated by local patient anatomy, the synthesized data can only highlight clinical associations and trends, rather than establish definitive causal factors for implant success. Currently, there are no universal recommendations for improving the effectiveness of orthodontic microimplant reinsertion. Further research is needed, including the evaluation of a broader range of standardized data, with an analysis of radiological examination results, highlighting the role of CBCT imaging.

Due to the high success rate of primary surgery, prospective studies are almost impossible, making it difficult to detect clinical protocol and medical record irregularities. Therefore, the use of computer models or biomechanical analyses should be considered to supplement clinical data.

Future research protocols and their associated evaluation methods should include detailed data such as:

- Information regarding the microimplant (manufacturer, model, material, length, diameter);
- Information regarding the etiology of the initial failure;
- The condition of the original implant and a detailed sterilization protocol (in the case of reuse of the original implant);
- Insertion torque and insertion angle;
- Insertion method (self-drilled vs. pre-drilled);
- Loading parameters (start time, force, direction);
- Radiological examinations (including assessment of cortical bone thickness and bone density);
- Mucosal type;
- Patient data (hygiene, general diseases, medications taken, type of defect);
- Clinical outcome of the reinsertion procedure (mobility, infections, time to loss).

## 5. Conclusions

Analysis of the literature regarding the success rate of orthodontic microimplant reinsertion procedures is currently subject to a high risk of interpretation errors. This results not only from the minimal amount of data contained in available publications, but, importantly, from the substantial risk of unmeasured confounding factors present in these non-randomized studies.

Furthermore, the evidence regarding the modification of the insertion site remains contradictory. While relocating the microimplant to areas with thicker cortical bone (such as the midpalatal suture) may offer biomechanical advantages, the current data do not universally confirm that changing the site provides significantly greater benefits than orthotopic reimplantation.

Due to the increasing adoption of these techniques to malocclusion treatment plans, it is advisable to continue standardized clinical and simulation studies to develop unified protocols for reinsertion procedures.

## Figures and Tables

**Table 1:** Inclusion and exclusion criteria.

Criteria	Inclusion	Exclusion
Population	Patients whose initial orthodontic microimplant procedure was unsuccessful and who required a repeat procedure (in vivo studies)	In vitro studies
Intervention	Reinsertion of the orthodontic microimplant	
Comparison	No comparison	
Outcomes	Reinsertion success rate and additional data (e.g., diameter, location, etc.)	
Settings	Clinical studies, cohort studies, case controls, case series	Letter, conference paper

**Table 2:** Data items with description.

Data Items	Description
Patient characteristics	Data were collected regarding the number, gender, and mean age of patients
Overall success of reoperation	The overall success rate of the reinsertion procedure was reported in percentage (%) and the proportions, due to the lack of standardization, ‘success’ was accepted as defined by the authors of the primary studies (e.g., stability over 8 months, 1 year, or until treatment completion).
Success based on implant location	To further characterize the problem, the success rate was reported in percentage (%) and the proportions for specific locations (maxilla vs. mandible; palatal suture area, anterior and lateral alveolar process)
Success based on whether or not repositioning was performed	The success rate was reported in percentage (%) and the proportions, depending on whether the reinsertion procedure was performed in the same or ectopic location compared to the initial procedure
Success based on implant characteristics	The success rate was calculated in percentage (%) and the proportions, depending on the length and diameter of the microimplant
Success based timing of microimplant reinsertion	The success rate was reported in percentage (%) and the proportions, depending on the time elapsed since the initial microimplant loss (immediately vs. 1 month vs. 2 months)

**Table 3:** Characteristics of included studies and implantation success rates.

Article	Number of Patients	M/F (Male/Female)	Number of Implants	Number of Successful Implants	Success Rate (%)	Mean Age	SD
Xin Y, 2025 [6]	71	9/62	100	66	66	26.12	7.07
Uesugi, 2018 [15]	94	20/74	94	60	63.83	N/A	N/A
Uesugi, 2017 [16]	77	17/60	77	34	44.16	N/A	N/A
Baek, 2008 [17]	34	17/17	34	21	61.76	21.78	5.85

N/A—not applicable.

**Table 4:** Success rates (SR) and number of implants divided into maxilla (Maxilla) and mandible (Mandible).

Article	Maxilla SR (%)	Successful Implants in the Maxilla	Number of Implants in the Maxilla	Mandible SR (%)	Successful Implants in the Mandible	Number of Implants in the Mandible
Xin Y, 2025 [6]	68.60	59	86	50	7	14
Uesugi, 2018 [15]	63.83	60	94	N/A	N/A	N/A
Uesugi, 2017 [16]	46.55	27	58	36.84	7	19
Baek, 2008 [17]	61.76	21	34	N/A	N/A	N/A

N/A—not applicable.

**Table 5:** Success rates (SR) for orthotopic and ectopic implantation.

Article	Orthotopic SR (%)	Successful Implants in the Same Localization	Number of Implants in the Same Localization	Ectopic SR (%)	Successful Implants in Different Localizations	Number of Implants in Different Localizations
Xin Y, 2025 [6]	66.67	42	63	64.86	24	37
Uesugi, 2018 [15]	61.33	46	75	73.68	14	19
Uesugi, 2017 [16]	45.76	27	59	38.89	7	18
Baek, 2008 [17]	68.42	13	19	53.33	8	15

**Table 6:** The influence of implant length on success rates (SR) and the number of successful procedures in the analyzed studies.

Article	5 mm SR (%)	Successful Implants	Number of Implants	6 mm SR (%)	Successful Implants	Number of Implants	8 mm SR (%)	Successful Implants	Number of Implants	10 mm SR (%)	Successful Implants	Number of Implants
Xin Y, 2025 [6]	N/A	N/A	N/A	N/A	N/A	N/A	67.03	61	91	55.56	5	9
Uesugi, 2018 [15]	N/A	N/A	N/A	51.35	19	37	71.05	27	38	N/A	N/A	N/A
Uesugi, 2017 [16]	N/A	N/A	N/A	28.89	13	45	65.63	21	32	N/A	N/A	N/A
Baek, 2008 [17]	61.76	21	34	N/A	N/A	N/A	N/A	N/A	N/A	N/A	N/A	N/A

N/A—not applicable.

**Table 7:** The influence of implant diameter on the success rate (SR) and the number of successful procedures.

Article	1.4 mm SR (%)	Successful Implants	Number of Implants	1.6 mm SR (%)	Successful Implants	Number of Implants	2.0 mm SR (%)	Successful Implants	Number of Implants
Xin Y, 2025 [6]	67.03	61	91	N/A	N/A	N/A	55.56	5	9
Uesugi, 2018 [15]	51.35	19	37	67.74	21	31	85.71	6	7
Uesugi, 2017 [16]	41.81	23	55	50.00	11	22	N/A	N/A	N/A
Baek, 2008 [17]	N/A	N/A	N/A	N/A	N/A	N/A	61.76	21	34

N/A—not applicable.

**Table 8:** Summary of clinical results depending on the implant loading protocol.

Article	Immediate	Successful Implants	Number of Implants	1 m. Latency	Successful Implants	Number of Implants	2 m. Latency	Successful Implants	Number of Implants
Xin Y, 2025 [6]	N/A	N/A	N/A	N/A	N/A	N/A	N/A	N/A	N/A
Uesugi, 2018 [15]	80	4	5	60.61	20	33	59.46	22	37
Uesugi, 2017 [16]	N/A	N/A	N/A	45.00	9	20	46.15	18	39
Baek, 2008 [17]	53.3	8	15	68.4	13	19	N/A	N/A	N/A

N/A—not applicable.

## Data Availability

All data are contained within this article.

## Supplementary Materials

The following supporting information can be downloaded at: https://www.mdpi.com/article/10.3390/jcm15093489/s1, File S1: PRISMA Abstract Checklist; File S2: PRISMA Checklist [32].

## Abbreviations

The following abbreviations are used in this manuscript:

TADs	Temporary Anchorage Devices
OSF	Open Science Framework
BASE	Bielefeld Academic Search Engine
CAD/CAM	Computer-Aided Design/Computer-Aided Manufacturing
K	Cohen’s kappa coefficient
SR	Success Rate
SD	Standard Deviation
CBCT	Cone Beam Computed Tomography
MI	Microimplant/Microimplant
CT	Cortical plate Thickness
BT	Total Bone Thickness
IT	Insertion Torque
MRI	Magnetic Resonance Imaging

## Appendix A

**Table A1:** Characteristics of microimplant systems used in the analyzed studies.

Article	System/Manufacturer	Type	Material	Size (mm)	Technique
Xin 2025 [6]	VectorTAS^®^ (Ormco) and Microimplant Anchorage^®^ (DentOS)	Self-drilling microimplants	Titanium alloy	Ø1.4 × 8.0 (Ormco)/Ø2.0 × 10.0 (DentOS)	Flapless, no pre-drilling
Uesugi 2018 [15]	Dualtop^®^; Jeil Medical, Seoul, Republic of Korea	Self-drilling, titanium microimplant	Titanium (Grade V, Ti–6Al–4V)	Ø1.4/Ø1.6 (MB) i Ø1.6/Ø2.0 (MP) × 6.0/8.0	Flapless, manual insertion
Uesugi 2017 [16]	Dualtop^®^; Jeil Medical, Seoul, Republic of Korea	Self-drilling, titanium microimplant	Titanium (Grade V, Ti–6Al–4V)	Ø1.4/Ø1.6 × 6.0/8.0	Flapless, manual insertion
Baek 2008 [17]	ORTHOplant^®^; BioMaterials Korea, Seoul, Republic of Korea	Self-drilling, conical shape	Titanium	Ø2.0 (upper) × 5.0	Flapless, 90° insertion to tooth axis

**Table A2:** Success rates (SR) of implants depending on the specific anatomical location.

Article	Palatal Area	Successful Implants	Number of Implants	Maxillary Posterior	Successful Implants	Number of Implants	Mandibular Posterior	Successful Implants	Number of Implants
Xin Y, 2025 [6]	N/A	N/A	N/A	69.05	58	84	50.00	7	14
Uesugi, 2018 [15]	88.9	16	18	58.33	42	72	N/A	N/A	N/A
Uesugi, 2017 [16]	N/A	N/A	N/A	46.43	26	56	36.84	7	19
Baek, 2008 [17]	N/A	N/A	N/A	61.76	21	34	N/A	N/A	N/A

**Table A3:** Excluded articles at the full-text screening stage.

Author, Year	Identificator	Reason
Arqub, 2021 [33]	10.2319/090720-777.1	Population
Kim, 2020 [34]	doi.org/10.3390/ma13194433	Population
